# Neural tissue engineering: From bioactive scaffolds and in situ monitoring to regeneration

**DOI:** 10.1002/EXP.20210035

**Published:** 2022-04-16

**Authors:** Bowen Gong, Xindan Zhang, Ahmed Al Zahrani, Wenwen Gao, Guolin Ma, Liqun Zhang, Jiajia Xue

**Affiliations:** ^1^ Beijing Laboratory of Biomedical Materials Beijing University of Chemical Technology Beijing China; ^2^ State Key Laboratory of Organic–Inorganic Composites Beijing University of Chemical Technology Beijing China; ^3^ Department of Mechanical and Materials Engineering University of Jeddah Jeddah Saudi Arabia; ^4^ Department of Radiology China–Japan Friendship Hospital Beijing China

**Keywords:** external stimulation, nerve guidance conduit, nerve imaging, peripheral nerve injury, tissue engineering

## Abstract

Peripheral nerve injury is a large‐scale problem that annually affects more than several millions of people all over the world. It remains a great challenge to effectively repair nerve defects. Tissue engineered nerve guidance conduits (NGCs) provide a promising platform for peripheral nerve repair through the integration of bioactive scaffolds, biological effectors, and cellular components. Herein, we firstly describe the pathogenesis of peripheral nerve injuries at different orders of severity to clarify their microenvironments and discuss the clinical treatment methods and challenges. Then, we discuss the recent progress on the design and construction of NGCs in combination with biological effectors and cellular components for nerve repair. Afterward, we give perspectives on imaging the nerve and/or the conduit to allow for the in situ monitoring of the nerve regeneration process. We also cover the applications of different postoperative intervention treatments, such as electric field, magnetic field, light, and ultrasound, to the well‐designed conduit and/or the nerve for improving the repair efficacy. Finally, we explore the prospects of multifunctional platforms to promote the repair of peripheral nerve injury.

## Graphical abstract

In this paper, we mainly introduce the recent progress on the construction of nerve conduit for the treatment of peripheral nerve injury, including the design of the conduits in combination with biological effectors and cellular components. In addition, we give perspectives on nerve imaging to allow for the in situ monitoring, and the application of intervention treatment means, such as electricity, magnetism, ultrasound, and light, to the well‐designed conduit and the nerve for promoting the treatment efficacy.

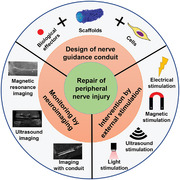

## 1 INTRODUCTION

Peripheral nerve injury (PNI) is a major medical issue that affects the sensory or motor ability after nerve disruption.^[^ ^1^ ^]^ For the peripheral nerve defect in a small gap (<5 mm), an end‐to‐end nerve suturing is often used clinically. It is relatively easy to perform but can lead to misaligned bridging of the proximal and distal nerve stumps, resulting in poor or even failed repair.^[^ ^2^ ^]^ When the gap is larger than 5 mm, a graft is necessary to bridge the two ends of the nerve stumps for restoring the nerve function. In this case, the “golden standard” in clinical treatment is autograft. However, this method suffers from drawbacks such as generation of a secondary injury, loss of function at the donor site, limited donor resources, and mismatch of the donor nerve with the injury.^[^ ^3^ ^]^

As an alternative, artificial nerve guidance conduits (NGCs) have been developed to provide a promising solution for PNI repair.^[^ ^3^ ^]^ Specifically, an ideal tissue engineered NGC can mimic the anatomical structure of a native nerve by combining the scaffold with biological effectors and cellular components. In general, NGCs can be fabricated from natural or synthetic materials by different methods, such as casting, electrospinning, and 3D printing.^[^ ^4^, ^5^, ^6^, ^7^ ^]^ Casting from a polymer solution to prepare NGCs has the advantages of simple process and easy industrialization.^[^ ^5^ ^]^ However, the wall of the obtained NGC is usually dense with a smooth surface and sometimes retains toxic organic solvents. To endow the wall of the conduit with physically oriented structures, other techniques can be integrated, such as photolithography to manufacture molds with specific structures, or post‐processing of the NGC (e.g., phase separation and freeze drying) to generate porous structures. Electrospinning represents another most used technique to fabricate NGCs composed of nanofibers.^[^ ^6^ ^]^ The alignment of the nanofibers in the wall of the conduit can be well regulated to manipulate cell behavior and promote nerve regeneration, and the porous structure is also beneficial for the exchange and transport of nutrients and wastes. However, the fiber diameter and the porosity are often distributed in a certain range, and the production efficiency of the conduit also needs to be further improved. In addition, 3D printing techniques, such as digital light processing‐ and extrusion‐based 3D printing, have been used for the preparation of NGCs.^[^ ^7^ ^]^ Digital light processing‐based 3D printing can rapidly produce single‐tubular or multi‐branched NGCs, but this technique is inhibited by the limited choice of available materials. Extrusion‐based 3D printing can print NGCs made of microfibers, allowing for the control of the structure with high reproducibility. However, it is difficult to produce nanoscale structures, and the resolution needs to be further improved. In addition, to fabricate a fibrous conduit in an aligned arrangement, support structures in the perpendicular direction will be often necessary, which may reduce the physical guidance effect to the cells and axons.^[^ ^4^ ^]^ The above techniques can sometimes be combined together to fabricate NGCs with well‐designed structures, such as 3D printed‐electrospinning method.

In recent years, great progress has been made in the design and construction of multifunctional tissue engineered NGCs by combining different types of guidance cues in one platform to facilitate the repair of nerve injury. According to the size of the gap, the design of the NGCs can be different. For the repair of a gap smaller than 5 mm, the design of the NGC is often simple because of the self‐regenerative capacity of the peripheral nerve. As for a larger gap over 5 mm, it will be necessary to provide multiple types of guidance cues by the NGC to promote the axon elongation and function recovery. Although some commercialized NGCs have been applied clinically, the repair efficacy is often limited due to their own structural and functional defects. A gap of about 30 mm is the critical length for a commercial NGC to repair the human nerve defects.^[^ ^8^ ^]^ When it comes to a thick nerve, the situation can be even worse. The development of NGCs that can effectively repair a thick nerve in large gap remains a great challenge. In general, the treatment efficacy of a NGC needs to be firstly evaluated by animal nerve injury models before a clinical trial, from small animals such as rats and rabbits to large animals like dogs, pigs, sheep, and non‐human primates.^[^ ^9^ ^]^ For the most commonly used in vivo rat model of sciatic nerve injury, the typical value of the gap is 10–15 mm. For evaluating the efficacy of NGCs in repairing defects exceeding 15 mm, in vivo models are usually performed in large animals with a typical gap value of 15–30 mm and even over 50 mm.^[^ ^10^ ^]^

The design of scaffolds with special architecture, the integration of biological effectors, and the incorporation of cellular components, as well as the intervention of externally physical stimuli have demonstrated therapeutic applications in nerve repair. Herein, we present the repair mechanism of peripheral nerve injuries and discuss the recent progress on the construction of tissue engineered NGCs for the treatment of PNI at small and large gaps. Afterward, we give perspectives on some efficient tools for in situ monitoring of the nerve repair process and key intervention treatment means to improve nerve repair efficacies from both clinical and research perspectives.

## 2 MECHANISM FOR THE REPAIR OF PNI

**FIGURE 1:**
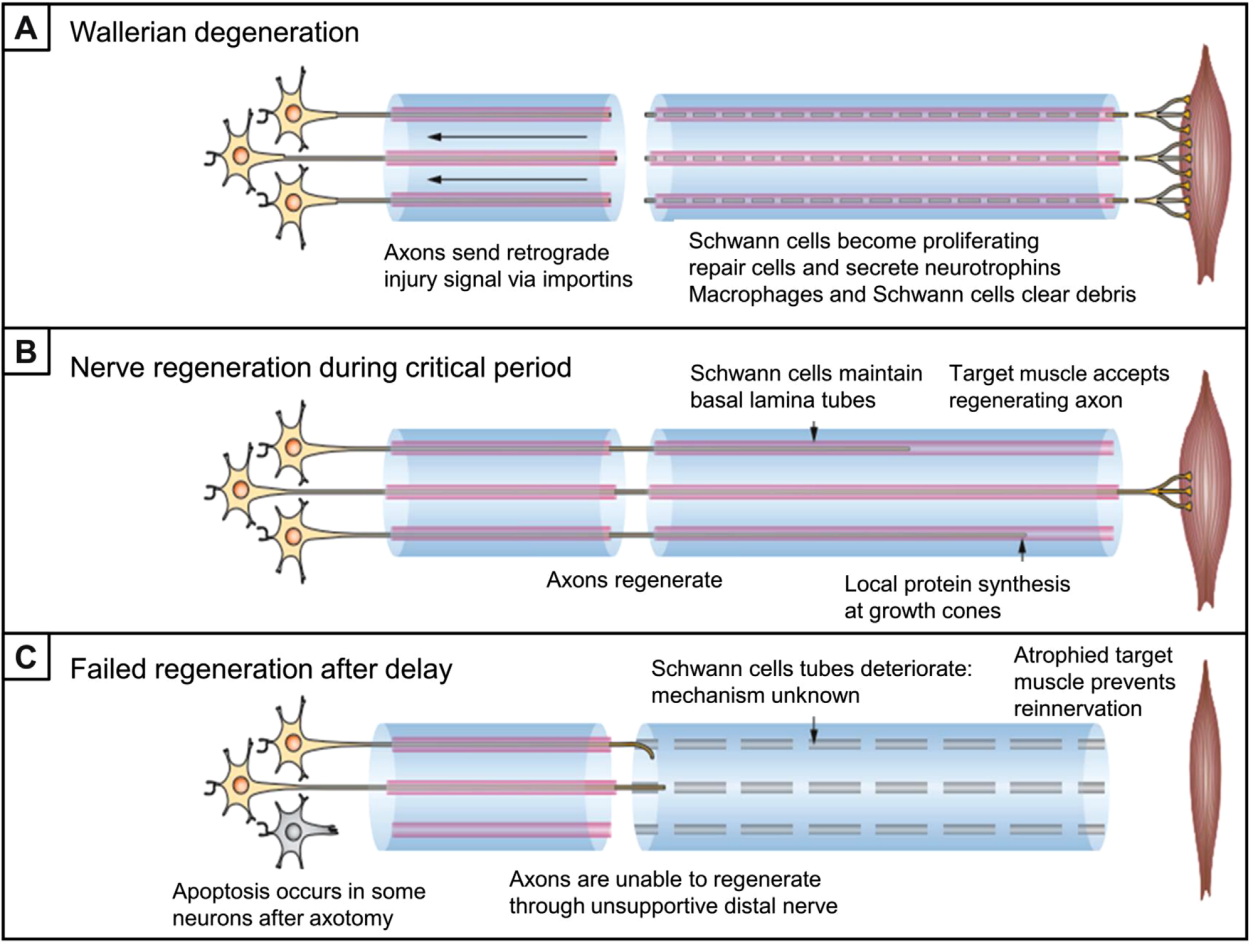
Schematic illustration showing the mechanism for the repair of peripheral nerve injury. (A) Wallerian degeneration and the associated behaviors of axons, SCs, and macrophages after the nerve injury. (B) Nerve regeneration during critical period. SCs maintain basal lamina tubes and local protein synthesize at growth cones of the axons to realize the regeneration of axons and ultimately the re‐innervation of the target muscles. (C) Any step out of order, such as the deterioration of SCs tubes, apoptosis of neurons, and failure of target muscle reinnervation, can result in the failure of regeneration. Reproduced with permission.^[^ ^11^ ^]^ Copyright 2013, Springer Nature

It is of great importance to understand the biological mechanism for the repair of PNI to guide and instruct the design of NGCs. After a nerve injury occurs, a series of sequential changes will happen at the injury site to provide a supporting microenvironment for nerve regeneration, as shown in Figure 1.^[^ ^11^ ^]^ Undergoing the Wallerian degeneration (Figure 1A), Schwann cells (SCs) dedifferentiate and restore the capability of proliferation while releasing chemokines and recruiting macrophages into the injured nerve site. The dedifferentiated SCs and recruited macrophages clean up the cell fragments produced by degeneration through endocytosis. At the same time, macrophages secrete vascular endothelial growth factor‐A (VEGF‐A) to promote macrovascular formation, which plays an important role in transporting nutrients and directing the directional migration of SCs.^[^ ^12^ ^]^ Afterward, the deposition and remodeling of the extracellular matrix (ECM) begin followed by the formation of Büngner bands based on SCs. SCs maintain the basal lamina tubes, providing a framework or a highway for axon regeneration.^[^ ^13^ ^]^ Meanwhile, the dedifferentiated SCs secrete various kinds of neurotrophic factors, such as nerve growth factor (NGF), brain‐derived neurotrophic factor (BDNF), neurotrophin‐3 (NT‐3), *etc*., promoting the axon extension.^[^ ^1^ ^]^ In addition, during axon extension, local protein synthesize at the growth cones of the axons, and multiple branched axons can be formed, but only the axons that correctly bridge with the distal nerve stump will be retained and reinnervate the target muscle. After a successful bridge is formed for the axons, the SCs will be remyelinated and the motor or sensory function will be partially restored (Figure 1B). If the axons fail to pass through the injured site and bridge the distal nerve, a neuroma will form, resulting in atrophy of the target tissue and the permanent loss of sensory or motor function (Figure 1C).^[^ ^11^ ^]^ Therefore, the several typical stages for nerve repair mainly include inflammation, angiogenesis, migration, and proliferation of SCs, extension of axons, and connection with target tissue.

For the nerve injuries with different sizes of gaps, the repairing mechanism is nearly the same, but it is obvious that the repair time will be extended in the case of larger gaps. A long‐term chronic denervation can lead to SCs atrophy, resulting in the degeneration and eventual disappearance of Büngner basement membrane.^[^ ^14^ ^]^ At the same time, atrophy also occurs to muscle tissue after a long‐term denervation. Therefore, the axons need to reach the target muscle tissue within a certain period of time, otherwise the muscle tissue will not be readily receptive to reinnervation.^[^ ^15^ ^]^ In the rodent PNI models, the favorable environment for nerve repair is usually maintained for only 4–8 weeks.^[^ ^16^ ^]^ At 8 weeks after injury, the ability of the distal nerve stump to support axon growth will be impaired, and axons can hardly grow into the distal nerve stump at 6 months.^[^ ^17^ ^]^ Therefore, it is particularly important to design NGCs that can facilitate the repair of nerve injury in a large gap that requires a long time for nerve bridging. The survival and activity of the neurons also largely affect the regeneration situation. The influence of axonal transection on rat neurons showed that apoptotic neurons could be found after 1 week of PNI.^[^ ^18^ ^]^ Another study showed that 35–40% of neurons in dorsal root ganglion (DRG) dead within 2 months after PNI.^[^ ^19^ ^]^ A similar phenomenon was also found in damaged nerves in humans.^[^ ^20^ ^]^ Therefore, it is the key to successfully repair large defects by shortening the nerve repair time, which can be realized by rapidly eliminating inflammation, enhancing angiogenesis, promoting proliferation and migration of SCs, and increasing the provision of neurotrophic factors to accelerate the extension of axons and remyelination of SCs.

## 3 NGCs FOR PERIPHERAL NERVE REPAIR

To overcome the current therapeutic barriers, an ideal NGC should not only have good biocompatibility and degradability but also prevent connective tissue penetration and support nerve regeneration facilitate partial or the entire repair process.^[^ ^21^ ^]^

### 3.1 NGCs for repairing nerve injury in small gap

For repairing nerve injury in a small gap (<5 mm), the clinical method of end‐to‐end neurorrhaphy may lead to the mis‐bridging of the nerve fibers. In this case, owing to the selective innervation of the native nerve fibers, a NGC can be applied to provide a favorable repair effect by serving as a regeneration chamber.^[^ ^22^ ^]^ The cavity in the conduit can avoid the tension to the nerve generated by suturing and provide a space for the autonomous and selective bridging of the proximal and distal axons. The convergence of selective reinnervation is more evident when it comes to a gap at a size of 2–4 mm.^[^ ^23^ ^]^ To a certain extent, the regeneration ability of the NGC can be better than the end‐to‐end neurorrhaphy. In this case, it may be a clinical trend to replace the end‐to‐end neurorrhaphy by NGC implantation to allow for the self‐recognition and selective innervation of the nerve fibers. There is a need to further improve the efficacy of NGC for the repair of nerve injury in small gap. The design of NGCs for repairing large gaps in the following section may also be applicable for a small gap.

### 3.2 NGCs for repairing nerve injury in large gap

To promote the repair outcome of nerve injury in large gap, tissue engineered NGCs have been designed and constructed by combining physicochemical cues, biological effectors, and/or cellular components, aiming to provide an ideal regeneration microenvironment. Although the nerve gap in animal injury models used in some studies is smaller than the critical repair length, the strategies can also provide some guidance for the design of the NGCs.

#### 3.2.1 NGCs with physicochemical cues

**FIGURE 2:**
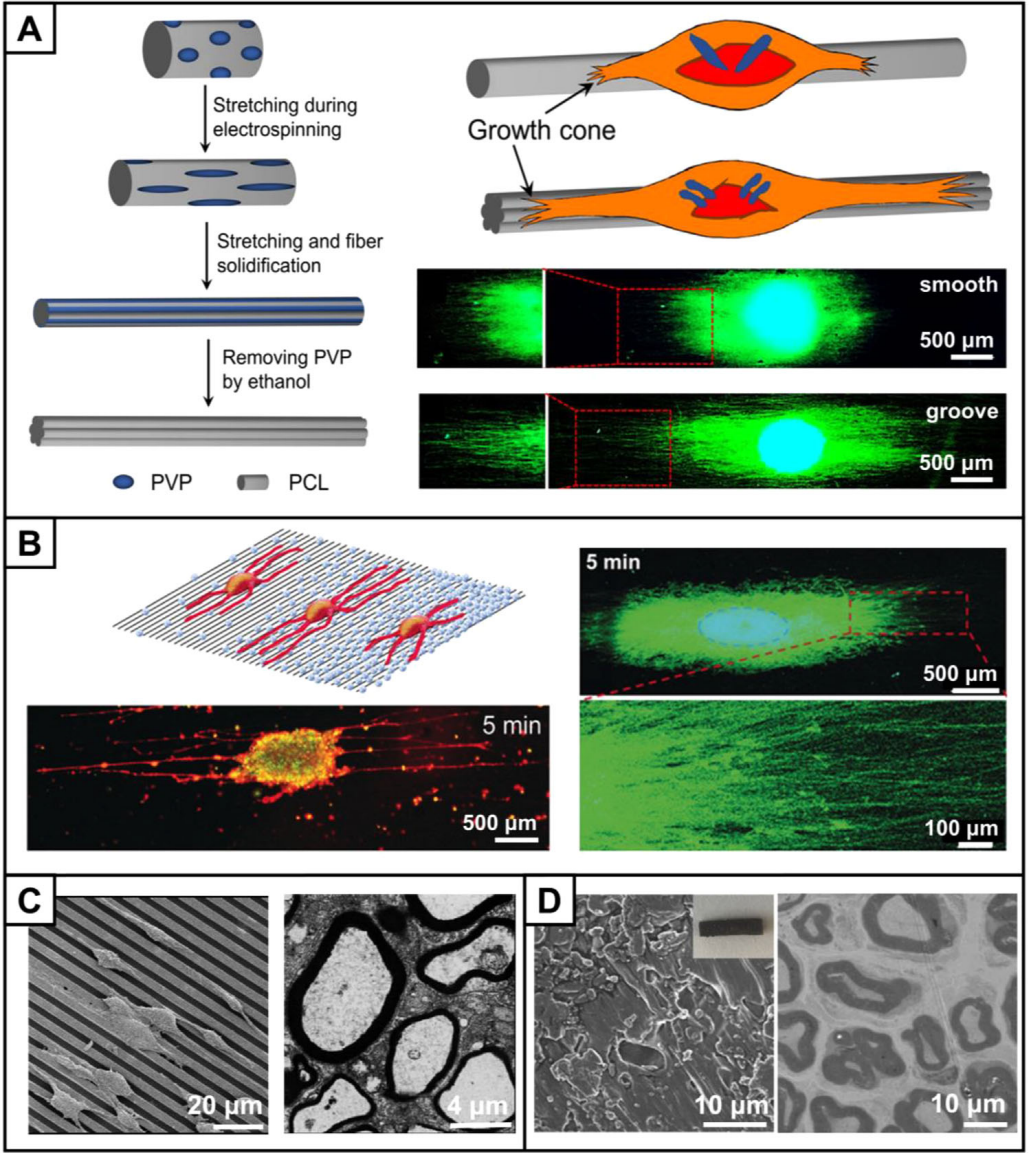
Construction of NGC with physicochemical cues. (A) Schematic illustrations showing the generation of nanoscale grooves on the surface of a microfiber (left). Schematic illustration (upper right) and fluorescence micrographs (lower right) showing the extension of neurites from DRG bodies cultured on the scaffolds with a smooth surface or with nanoscale grooves, respectively. The neurites from DRG bodies were stained with Tuj1 (green), and the cell nuclei were stained with 4′,6‐diamidino‐2‐phenylindole (DAPI, blue). Reproduced with permission.^[^ ^27^ ^]^ Copyright 2020, John Wiley & Sons. (B) Schematic illustration (upper left) indicating the extension of neurites after cultured on uniaxially aligned electrospun fibers deposited with microparticles at different densities, and fluorescence micrographs showing the neurites extension of a PC12 multicellular spheroid (lower left) and DRG (right), respectively, after cultured on uniaxially aligned fibers deposited with microparticles for 5 min. The PC12 multicellular spheroid was stained using Neurite Outgrowth Staining Kit. The yellow color corresponds to an overlay of the red and green fluorescence from the cell membrane and cell viability indicators, respectively. The neurites from DRG body were stained with Tuj1 (green), and the cell nuclei were stained with DAPI (blue). Reproduced with permission.^[^ ^28^ ^]^ Copyright 2019, John Wiley & Sons. (C) Scanning electron microscopy (SEM) image of macrophages cultured on the micropatterned conductive PLCL/GO film (left), and TEM image of the nerve regenerated in the micropatterned conductive conduit in a 10‐mm rat sciatic nerve injury model after 8 weeks (right). Reproduced with permission.^[^ ^30^ ^]^ Copyright 2020, The American Chemical Society. (D) SEM image (left) and the inserted optical image of the PCL/GO conduit, and TEM image of the nerve regenerated in the PCL/GO conduit in a 15‐mm rat sciatic nerve injury model after 18 weeks (right). Reproduced with permission.^[^ ^31^ ^]^ Copyright 2018, John Wiley & Sons

When designing a NGC, physicochemical cues can be integrated into the wall and/or lumen of the conduit to manipulate the different nerve repairing stages. The surface topography of the scaffold greatly affects the inflammation stage and cell behavior. For example, aligned structures are often applied for constructing the tube wall. Electrospun aligned fibers could convert macrophages from pro‐inflammatory M1 to anti‐inflammatory M2 phonotype.^[^ ^24^ ^]^ In combination with deferoxamine, an angiogenic reagent, electrospun aligned fibers could promote angiogenesis while regulating the phenotype of macrophages.^[^ ^25^ ^]^ A suitable regenerative immune microenvironment was thus constructed because of the reduction of inflammatory factors and the formation of blood vessels, accelerating the nerve repair in a 10‐mm rat sciatic nerve injury model. The directional structures also have a significant effect on SCs migration and axon extension, which can also be combined with other nano‐ or micro‐cues.^[^ ^26^ ^]^ For example, nanogrooves with a width of about 200–300 nm were generated on individual fibers by electrospinning, which could maximize the guidance of neurite extension and SCs migration by providing more contact area and induction for the neuronal growth cones (Figure 2A).^[^ ^27^ ^]^ The surface roughness of the substrate also affects the neurite extension. Electrospun aligned fibers decorated with electrosprayed fatty acid microparticles with a moderate density could endow the surface of the scaffold with optimal surface roughness to promote neurites extension from both spheroid of PC12 cells and DRG (Figure 2B).^[^ ^28^ ^]^

Electrical signal represents a specific type of physical cue that can be integrated into the NGC because of the electrical activity of nerve. Conductive materials, such as graphene oxide (GO), gold, and conductive polymers, can be combined to upgrade the conduits.^[^ ^29^ ^]^ In one study, an electrically conductive poly (l‐lactide‐*co*‐caprolactone) (PLCL)/GO film with micropatterns could regulate the phenotype of macrophages and promote the in vivo vascularization, SCs migration, and remyelination to repair a 10‐mm rat sciatic nerve injury (Figure 2C).^[^ ^30^ ^]^ As shown in the transmission electron microscopy (TEM) image, the average thickness of the regenerated myelin sheath and the average diameter of the regenerated axons in the micropatterned conductive conduit were about 0.45 and 3 μm respectively, after 8 weeks, which were significantly larger than those in a flat conduit group, indicating that the nerve regeneration was significantly promoted. In another study, conductive GO was incorporated in polycaprolactone (PCL) to fabricate a conductive conduit for repairing a 15‐mm rat sciatic nerve injury (Figure 2D).^[^ ^31^ ^]^ From the TME image, the average thickness of the regenerated myelin sheath and the average diameter of the myelinated axons in the conductive conduit were 2.5 and 6 μm, respectively, after 18 weeks, which were similar to those in the autograft group, indicating that the conduit achieved a regeneration efficacy comparable to the autograft. The proper π‐π bonding in GO contributed to cell bioelectricity and improved the metabolic activity of cells.^[^ ^32^ ^]^ A long‐term evaluation of conductive scaffolds in vivo is necessary because of the biosafety concern.

As an efficient strategy, NGCs can be filled with materials such as gels, sponges, fibers, and yarns in the lumen to provide a further topographical support and induction for cell growth and axon extension.^[^ ^33^, ^34^ ^]^ A conduit filled with oriented fibers could promote the polarization of macrophages to the M2 phenotype and the migration and proliferation of SCs, as well as the axon extension.^[^ ^33^ ^]^ These filling materials can also be used as carriers of biological effectors and exogenous cells. Another efficient strategy is integrating multiple channels in the conduit to mimic the fascicle structure of peripheral nerve, which is beneficial for increasing the contact area of cells and axons with the conduit. A multi‐tubular conduit was constructed by simply wrapping three or seven small tubes into a larger tube.^[^ ^35^ ^]^ Other methods, such as template molding and 3D printing have also been applied to prepare multi‐channel conduits with typical size and tube number.^[^ ^36^ ^]^ The problem faced by the multi‐channel conduit is that the number and diameter of the channels need to be optimized to adapt for the variable anatomical structures of the nerve bundles in different locations.

Taken together, scaffolds integrated with physicochemical cues can play a regulatory role on cell behavior and axon extension. These cues generally include oriented topographic structure, specific patterns, appropriate surface roughness, multi‐channel structures, and so on. For promoting PNI repair, the role of physicochemical cues is specifically manifested in regulating the phenotype of macrophages, accelerating the migration of SCs, manipulating the differentiation of exogenous stem cells, and guiding the extension of axons. Endowing the scaffold with a special structure is only the first step in the long journey. To further facilitate the repair of injuries in large gap, biological cues are often necessary in the scaffolds to provide a more favorable environment.

#### 3.2.2 NGCs with biological effectors

Peripheral nerve regeneration involves the interaction of multiple types of biological effectors, such as neurotrophins and bioactive proteins. They can be loaded in or immobilized on the wall of the tube or in the microspheres, gels, and fibers filling in the lumen of the NGC, to manipulate cell behavior and axon extension.^[^ ^37^ ^]^

In the early stage of nerve injury, it is necessary to provide biological effectors capable of suppressing the inflammatory response. For example, melatonin has the functions of scavenging free radicals, inhibiting cell apoptosis, and preventing scar formation. A NGC loaded with melatonin successfully repaired a 15‐mm sciatic nerve injury in a rat model.^[^ ^38^ ^]^ Both the thickness of the regenerated myelin sheath and the nerve conduction velocity were even higher than those in the autograft group. The deposition of large amounts of chondroitin sulfate proteoglycans (CSPGs) can lead to the formation of scar, inhibiting the axon extension. In this regard, typical types of enzymes or proteins can be applied to elaborate or even avoid scar formation. For example, chondroitinase ABC could be encapsulated in a NGC composed of electrospun fibers to induce the degradation of CSPG.^[^ ^39^ ^]^

**FIGURE 3:**
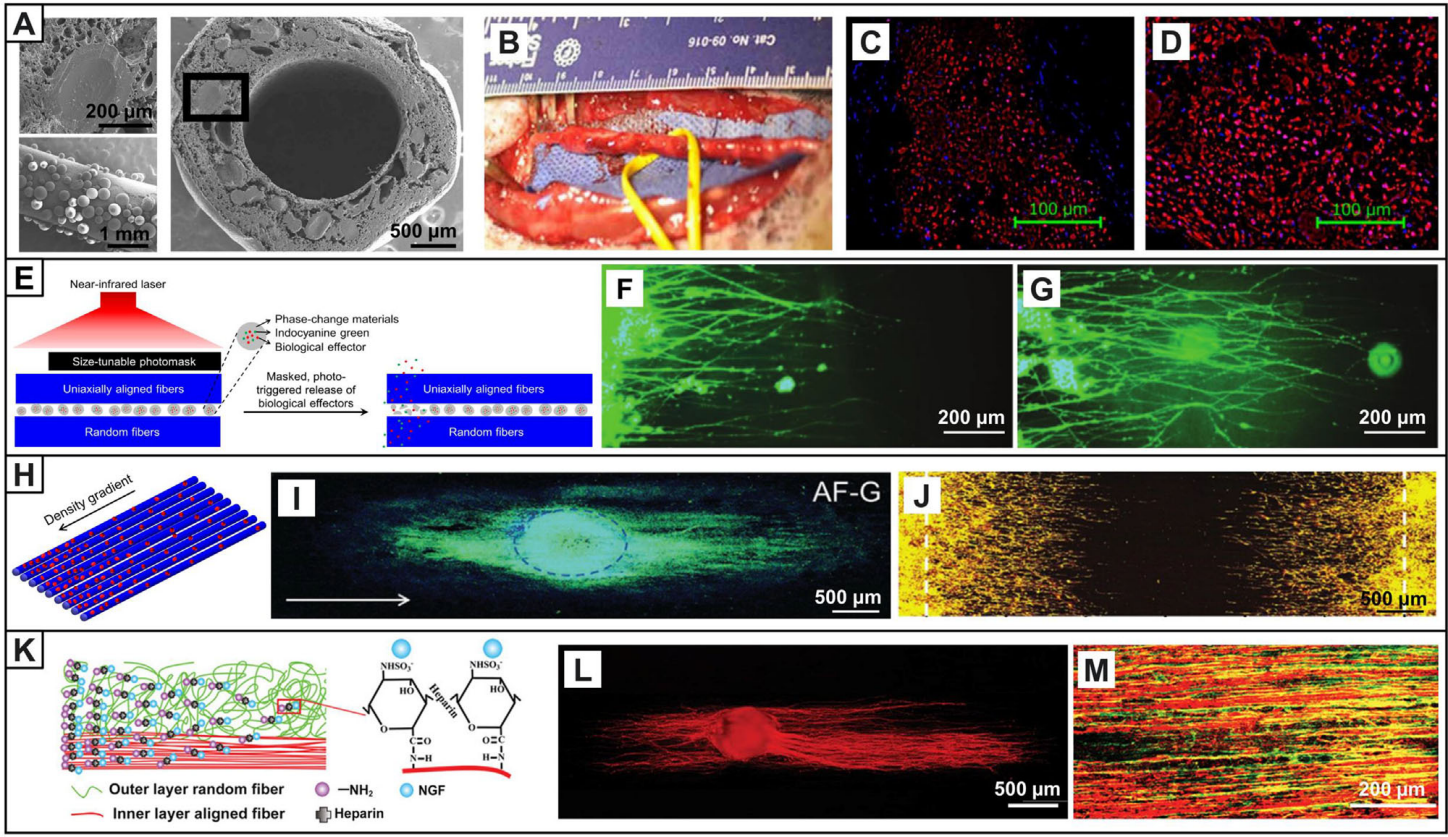
NGCs combined with biological effectors to regulate cell behavior and neurites extension. (A) SEM images showing that PLGA microspheres‐encapsulated with GDNF were loaded in the wall of a PCL conduit. (B) Digital image of the conduit containing GDNF after implanted for 1 year to repair a 5‐cm median nerve defect in a rhesus monkey model. Fluorescence micrographs showing SCs in the distal nerves repaired by (C) the autograft and (D) the conduit containing GDNF, respectively. The SCs were stained with S100 (red), and the cell nuclei were stained with DAPI (blue). Reproduced with permission.^[^ ^40^ ^]^ Copyright 2020, The American Association for the Advancement of Science. (E) Schematic illustration showing the photo‐triggered release of biological effectors from a scaffold by irradiation with a near‐infrared laser and a size‐tunable photomask. Fluorescence micrographs showing the typical neurites extending from spheroids of PC12 cells cultured on scaffold under (F) single exposure and (G) masked irradiation, respectively. The PC12 cells were stained with Tuj1 (green), and the cell nuclei were stained with DAPI (blue). Reproduced with permission.^[^ ^43^ ^]^ Copyright 2020, John Wiley & Sons. (H) Schematic illustration showing the distribution of nanoparticles on uniaxially aligned nanofibers in a unidirectional density gradient. (I) Fluorescence micrograph showing the neurites extending from DRG bodies on the graded scaffold. The white arrowed line indicates the direction of increasing the density of the nanoparticles. The neurites from DRG bodies were stained with Tuj1 (green), and the cell nuclei were stained with DAPI (blue). (J) Fluorescence micrograph showing the migration of SCs from the two ends toward the center on the graded scaffold on which the density of nanoparticles increased from the two sides to the center. The actin cytoskeleton and the vinculin were stained with Alexa Fluor 555 phalloidin (red) and Alexa Fluor 488 anti‐vinculin (green), respectively, and the yellow color corresponded to an overlay of these two colors. Reproduced with permission.^[^ ^45^ ^]^ Copyright 2020, John Wiley & Sons. (K) Schematic illustration showing the fabrication of a NGF‐gradient/aligned PCL fibrous scaffold. (L) Neurites extension from the DRG (red) cultured on the NGF‐gradient/aligned PCL fibrous scaffold. (M) Fluorescence micrograph showing the morphology of the regenerated nerve in the middle portion of the NGF‐gradient/aligned PCL fibrous conduit after repairing a 15‐mm rat sciatic nerve injury for 12 weeks. Neurofilaments were stained with NF‐200 (green), and SCs were stained with S100 (red). Reproduced with permission.^[^ ^46^ ^]^ Copyright 2020, John Wiley & Sons

After the inflammatory response is eliminated, growth factors, such as VEGF and fibroblast growth factor (FGF), can be delivered to promote vascularization for providing structural support and nutrients for subsequent cell activities. Some additional biological effectors, such as neurotrophic factors, polypeptides, ECM components, and exosomes, also play an important role in nerve regeneration. In one study, poly (lactic‐*co*‐glycolic acid) (PLGA) microspheres‐encapsulated with glial‐derived neurotrophic factor (GDNF) were loaded in the wall of a PCL conduit (Figure 3A) and then implanted to repair a 5‐cm median nerve defect in a rhesus monkey model (Figure 3B).^[^ ^40^ ^]^ As shown in the fluorescence micrographs in Figure 3C,D, compared to that using an autograft (Figure 3C), a higher fluorescence intensity of SCs was observed in the distal regenerated nerve using the NGC containing GDNF (Figure 3D), demonstrating a significant improvement in the repair effect. A functional recovery comparable to the autograft group was also achieved. It is ideal to realize a synergistic or sequential release of typical types of biological effectors at the different regeneration stages. For example, NGF and BDNF were sequentially delivered from a multi‐channel NGC.^[^ ^41^ ^]^ The conduit could successfully repair a 15‐mm nerve defect in rabbit, in which the early delivery of NGF promoted axon regeneration in the initial stage while the delayed release of BDNF enhanced the late stage of myelination. The effectively physiological concentration ranges of biological effectors in vivo are worth of further investigation to avoid unexpected risk and side effects.

A triggered release of biological effectors is important for improving their utilization efficiency and realizing a continuous and on‐demand provision at specific time post‐surgery. Non‐invasive external stimulations, such as near‐infrared light and ultrasound, are promising triggers for achieving the spatiotemporally controlled release. In one study, microparticles made of phase‐change materials were encapsulated with NGF and indocyanine green and then integrated between two layers of electrospun fibers with the top layer made of uniaxially aligned fibers.^[^ ^42^ ^]^ Upon a near‐infrared laser irradiation, the phase‐change particles underwent solid‐liquid transition, allowing the triggered release of the encapsulated NGF, promoting the neurites extension. When a size‐tunable photomask was further introduced between the laser and the scaffold, a spatiotemporally controlled release of NGF was achieved (Figure 3E).^[^ ^43^ ^]^ Compared with a single exposure to the light to trigger the release of all the encapsulated NGF (Figure 3F), multiple irradiations at specific positions at different times (Figure 3G) could selectively and gradually trigger the release of NGF, accelerating the neurites extension of DRG. In the microparticles, specific types of biological effectors can be loaded and then selectively positioned in the conduit, allowing the triggered release of desired effectors at specific time to match the regeneration stages and provide an ideal microenvironment.

The concentration gradient of biological effectors also affects cell behavior and axon extension because of chemotaxis and/or haptotaxis effects.^[^ ^44^, ^45^, ^46^ ^]^ A unidirectional density gradient of nanoparticles made of a mixture of collagen and fibronectin were deposited by electrospraying on uniaxially aligned fibers (Figure 3H), promoting the neurites extension of DRG along the direction of increasing the density of the mixture (Figure 3I).^[^ ^45^ ^]^ A bidirectional gradient of the particle density on the scaffold promoted the migration of SCs from the two sides with a low‐density deposition toward the center with a high‐density deposition (Figure 3J). In addition to bioactive proteins, a gradient of growth factors can also be generated to guide directional cell migration and neurite extension. By generating a gradient of NGF on an oriented scaffold through heparin (Figure 3K), the neurites extension of DRG in vitro was significantly promoted along the direction of increasing the concentration (Figure 3L).^[^ ^46^ ^]^ After repairing a 15‐mm sciatic nerve injury in rat for 12 weeks, the sciatic nerve index and the compound muscle action potential (CMAP) of the rats implanted with the graded conduits were similar to that with the autograft, and the regenerated nerve fibers were highly oriented (Figure 3M).

Integrating biological effectors in scaffolds is an effective way for repairing PNI. The positive effects of the biologically active scaffolds on nerve repair are specifically manifested in the elimination of reactive oxygen radicals, inhibition of early inflammatory response and scar formation, facilitation of angiogenesis, acceleration of SCs migration and proliferation, and promotion of axon extension.

#### 3.2.3 Scaffolds with cellular components

**FIGURE 4:**
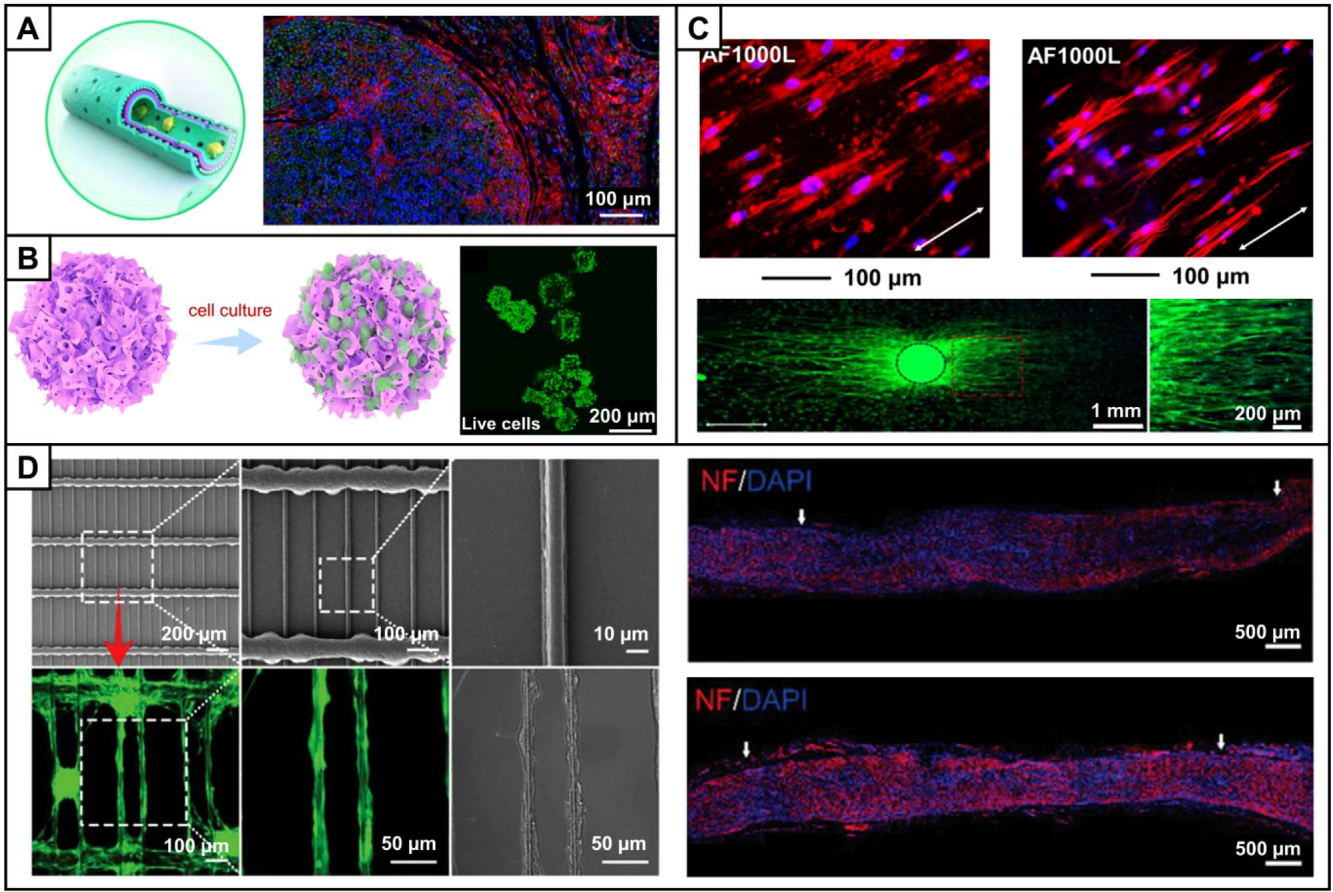
(A) Schematic illustration showing the structure of the SCs‐loaded polydopamine/arginylglycylaspartic acid‐multilayered graphene/PCL NGC, and the immunofluorescence micrograph showing the regenerated nerve stained with Tuj1 (green), NF200 (red), and DAPI (blue). Reproduced with permission.^[^ ^32^ ^]^ Copyright 2018, Springer Nature. (B) Schematic illustration of ADSCs‐laden polylysine‐decorated chitosan microcarriers, and the fluorescence micrograph of live cells (green) on the microspheres. Reproduced with permission.^[^ ^49^ ^]^ Copyright 2021, Elsevier. (C) Fluorescence micrographs of cells transdifferentiated from BMSCs on laminin‐coated aligned fibers with an average diameter of 1000 nm (AF1000L). The differentiated cells were stained with S100 (red, upper left) and Alexa Fluor 555 phalloidin (red, upper right), respectively, and the cell nuclei were stained with DAPI (blue). Fluorescence micrographs of the typical neurite fields extending from DRG cultured on the AF1000L scaffold. The neurites from DRG bodies were stained with Tuj1 (green), and the cell nuclei were stained with DAPI (blue). Reproduced with permission.^[^ ^51^ ^]^ Copyright 2017, American Chemical Society. (D) SEM and fluorescence micrographs (left) showing the structure of the 3D printed scaffold and the growth of NCSCs (green) on the scaffold, respectively. Fluorescence micrographs of the nerves regenerated in the scaffold (upper right) and NCSC‐loaded scaffold (lower right), respectively, in a 6‐mm rat sciatic nerve injury model. The regenerated nerves were stained with neurofilament (NF, red), and the cell nuclei were stained with DAPI (blue). Arrows indicate the start and end points of the regenerated nerve. Reproduced with permission.^[^ ^52^ ^]^ Copyright 2021, John Wiley & Sons

Cellular components represent another important cue for constructing tissue engineered NGCs. After nerve injury, a large number of SCs are required to migrate to and proliferate at the injury site in time to form the myelin sheath outside the axon and secret a variety of neurotrophic factors. In addition to inducing the migration of endogenous SCs, exogenous cells including SCs, stem cells, or stem cell‐derived‐SCs can be introduced to promote nerve regeneration. SCs or stem cells can be incorporated with NGCs by different ways, such as by directly injecting into the lumen of the conduit, by loading on other carriers such as gels and microspheres, and/or by seeding on the inner surface of the wall of the conduit.^[^ ^32^ ^]^ In the treatment of nerve injury, SCs play a crucial role in the whole process of nerve regeneration, involving in the regulation of nerve regeneration by secreting regeneration‐related factors and participating in the remyelination. As shown in Figure 4A, a porous multilayer conductive scaffold loaded with SCs could successfully promote axon regeneration and remyelination in repairing a 15‐mm rat sciatic nerve injury.^[^ ^32^ ^]^ In another study, autologous SCs were directly injected into a conduit to repair a 7.5‐cm human sciatic nerve injury, with partial return of sensory and motor functions were recovered after 15 months.^[^ ^47^ ^]^ SCs can also be functionalized prior to implantation to achieve specific function. For example, SCs that were induced to overexpress VEGF‐A by lentivirus‐mediated transfection could facilitate angiogenesis and thus promote nerve repair in a 10‐mm rat sciatic nerve injury model.^[^ ^48^ ^]^

The clinical transplantation of autologous SCs still faces some challenges due to their limited availability, invasive manipulation during cell collection, long culture time, and injury to the donor site. In this case, stem cells can be applied as an alternative. In one study, adipose‐derived stem cells (ADSCs) were carried by chitosan spheres (Figure 4B) and then loaded into a conduit, significantly promoting the nerve repair in a 10‐mm rat sciatic nerve injury model.^[^ ^49^ ^]^ However, the repair efficacy of the conduit was not as good as that of autograft, which might be because ADSCs only served to secrete regeneration‐related factors rather than differentiated into SCs. To improve the effect of stem cell therapy, the migration and differentiation of stem cells can be manipulated by engineering the surface chemistry, morphology, and structure of the scaffolds, as well as by incorporating biochemical and electrochemical signals.^[^ ^50^ ^]^ Electrospun aligned fibers with appropriate properties could effectively induce the differentiation of BMSCs to SCs after culturing in a differentiation medium, promoting the neurites extension of DRG (Figure 4C).^[^ ^51^ ^]^ Neural crest stem cells (NCSCs) were differentiated into SCs on a 3D printed scaffold, leading to the formation of thicker myelin sheath with a higher density in a 6‐mm rat sciatic nerve injury model (Figure 4D).^[^ ^52^ ^]^ It is important to manipulate the differentiation route of the incorporated cells, clarify the differentiation mechanism, and guide the cells to desired phenotype to improve cell survival, safety, and efficacy. Prior to in vivo implantation, the NGCs with cellular components can also be pre‐cultured in a bioreactor containing typical combinations of nutrients or using cyclic mechanical stimulation to regulate cellular phenotypes, which will be beneficial for accelerating the in vivo regeneration of axons and promoting the nerve repair outcome.^[^ ^53^, ^54^, ^55^ ^]^ In addition, cryo‐bio‐printing is an interesting method for preparing tissue engineered scaffolds with cells that can be cryopreserved and resuscitated while preserving the cell activity for subsequent implantation.^[^ ^56^ ^]^

The delivery of cells through a biologically active nerve conduit solves the deficiency of direct injection. In addition, the scaffold and cells can play a synergistic role together by regulating the behavior of the cells through the scaffold and allowing the cells to further integrate with the host tissue, ultimately promoting nerve regeneration. Tissue engineered NGCs are promising repair methods by effectively combining the above‐mentioned designs, which is promising to realize the repair of large defect in thick nerve. However, the efficient integration of the scaffolds, stem cells, and growth factors is still a challenge, and the translation of this type of conduit to clinical applications still has a long way to go.

## 4 PERSPECTIVES

Current research on scaffolds for PNI repair has mainly focused on the structural design and functional modification of the NGCs. After the conduit is implanted in vivo, the progress of nerve regeneration is often difficult to be observed and monitored in time. Combining the monitoring of the nerve repair process with appropriate postoperative intervention treatments is a promising way to further promote the nerve repair with a controllable fashion. In this case, imaging techniques can be applied to observe and detect the progress of nerve repair in real time, and then appropriate external stimulation (e.g., electric field, magnetic field, light, ultrasound, *etc*.) can be selectively applied at a controlled intensity to the well‐designed conduit.

### 4.1 In situ monitoring of the peripheral nerve repair process

**FIGURE 5:**
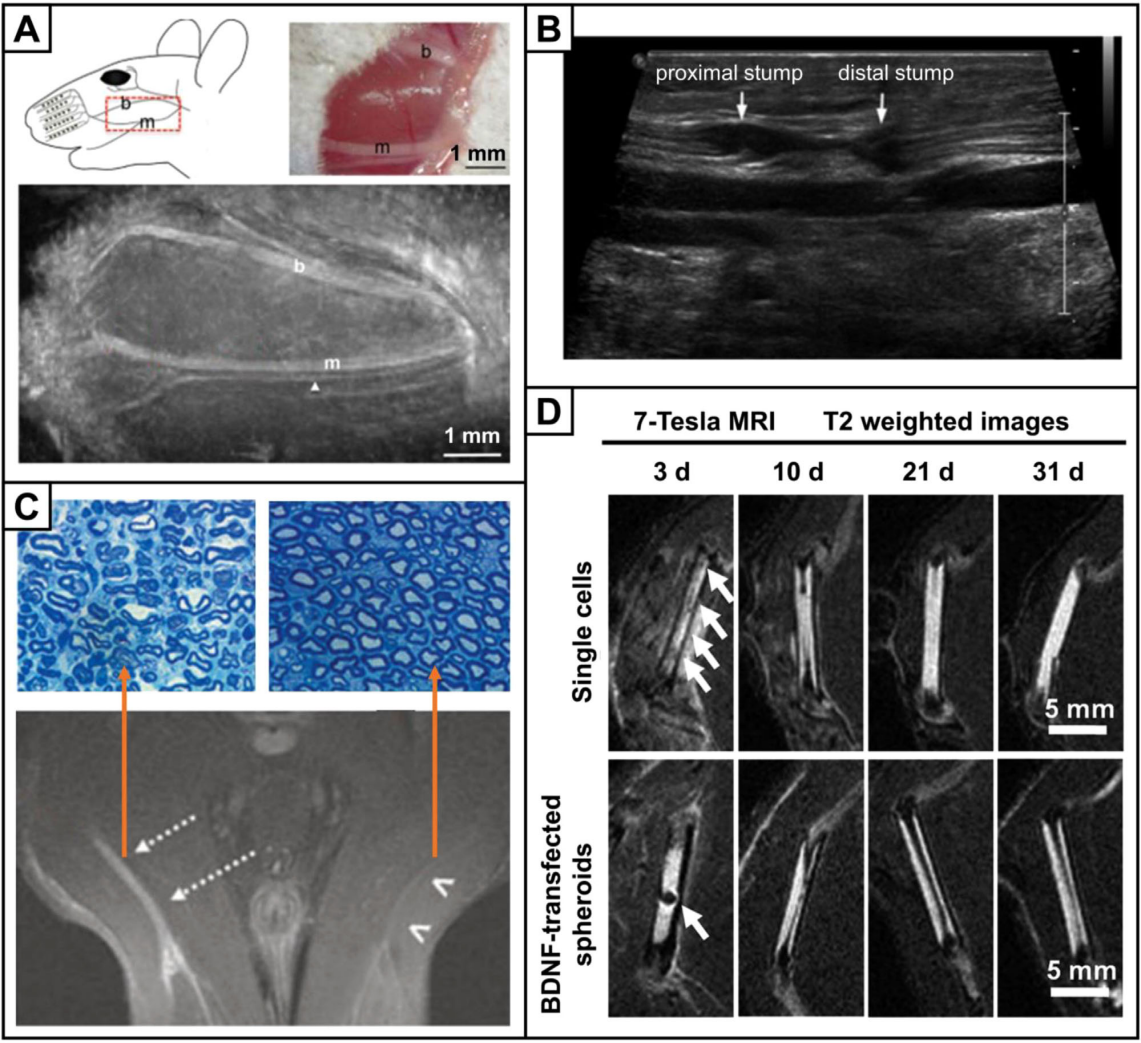
(A) MRI of the mouse facial nerve including the buccal (b) and marginal (m) branch. Reproduced with permission.^[^ ^57^ ^]^ Copyright 2019, Frontiers. (B) High‐resolution ultrasonography image of a 3‐cm transected tibial nerve (arrows) with neuroma at the proximal and distal stump margins. Reproduced with permission.^[^ ^58^ ^]^ Copyright 2015, American Association of Neurological Surgeons. (C) Differences in myelin and Gadofluorine M deposition at the damaged (dotted arrows) and undamaged sciatic nerve (arrowheads). Reproduced with permission.^[^ ^60^ ^]^ Copyright 2005, John Wiley & Sons. (D) Sagittal views of MRI of a 10‐mm rat sciatic nerve receiving conduits loaded with MSC single cells or BDNF‐transfected MSC spheroids at 3, 10, 21, and 31 days, respectively. Arrows indicate the Fe_3_O_4_ nanoparticles‐labeled cells. Reproduced with permission.^[^ ^68^ ^]^ Copyright 2014, Elsevier

Imaging techniques, such as magnetic resonance imaging (MRI) and ultrasound imaging, are often used clinically for the diagnosis of PNI. MRI is very sensitive to the proton composition of different tissues, which can be applied to induce a contrast within the nerves, as shown in Figure 5A, but it is difficult to clearly indicate the axonal damage or regeneration in the endoneurium at a high resolution.^[^ ^57^ ^]^ Ultrasound has been used to estimate the preserved and damaged fascicles in torn nerves, and the resolution also needs to be improved. As shown in Figure 5B, even in high‐resolution ultrasonography, it is difficult to clearly observe the transected tibial nerve and neuroma formation at the proximal and distal stumps.^[^ ^58^ ^]^ Other imaging technologies also have their own drawbacks in terms of the resolution.^[^ ^59^ ^]^

Visualizing the complex structures of peripheral nerves is one of the necessary conditions for an effective diagnosis of the nerve injury. Different types of imaging contrast agents have been developed to improve the imaging resolution. For example, GadoFluine M, a gadolinium (III) reagent, can accumulate in fibrous ECM components and degenerated myelin sheaths in the damaged sciatic nerves to achieve an improved imaging resolution (Figure 5C).^[^ ^60^ ^]^ Manganese chloride contrast agents have different affinities to nociceptive and non‐nociceptive nerves.^[^ ^61^ ^]^ However, currently there is still no clinically approved contrast agent that can selectively target to the peripheral nerves.^[^ ^62^ ^]^

Targeted imaging has made a considerable progress in other fields, which can provide guidance for the 3D reconstruction of nerves at the fascicle‐ and even axonal‐level. Ideal neuroimaging agents should have selective affinity to peripheral nerve‐associated cells or myelin sheaths, such as the axonal growth cone marker transient receptor potential channel 1 (TRPC1),^[^ ^63^ ^]^ short transient receptor potential channel 3(TrpC3), and neuronal damage markers.^[^ ^64^ ^]^ The increased expression of SCs surface marker p75 at the initial stage of injury will return to a lower level after axonal regeneration, which may also be an option for monitoring the repair process.^[^ ^65^ ^]^ The imaging agents can be combined with NGCs to realize a real‐time visualization of nerves in vivo. In this case, neuroimaging can help predict and give an early warning of unfavorable regeneration conditions in advance, such as axonal derailment, and then pre‐emptive intervention can be provided to reduce the failure rate.

The imaging agents can be encapsulated in the wall or the filling materials of the NGC and then release to selectively incorporate into the surrounding nerve tissue to help visualize the nerve regeneration. One approach is to encapsulate the imaging agents in the wall of the NGC and then release by degradation of the conduit or by diffusion. For example, iron oxide nanoparticles could be embedded in the conduit to enhance the contrast resolution under MRI.^[^ ^66^ ^]^ Perfluorocarbon‐labeled tissue scaffolds could also be imaged using ^19^F‐MRI.^[^ ^67^ ^]^ After the contrast agents are released from the conduit and enter the peripheral nerve tissue, the resolution of imaging can be improved to a certain extent. A long‐term imaging capability is preferable, which may be achieved by regulating the loading method and the interaction between the agents with the conduit. Imaging agents can also be released from NGCs due to the breaking of chemical bonds by molecules involved in the nerve regeneration process, such as certain matrix metalloproteinases or phospholipase A2.^[^ ^59^ ^]^ In this case, the change in the intensity of the imaging signal with time can also reflect the regeneration activity. The increased binding between the contrast agent and NGCs may prolong the imaging time, but the safety of long‐lasting contrast agents in vivo needs to be considered. Another approach is to embed the contrast agents in a filling material such as hydrogel in the lumen of the conduit. As the regenerated nerve tissue swells and the gel degrades, the contrast agents will be released from the matrix and enter the regenerated nerve for neuroimaging. Cells can also be used as a carrier of the contrast agent to achieve treatment and detection capabilities at the same time. In one study, individual MSCs or BDNF‐transfected MSC microspheres were used as the carrier of Fe_3_O_4_ nanoparticles to treat nerve injury and monitor the repair process.^[^ ^68^ ^]^ As shown in Figure 5D, the BDNF‐transfected stem cells could complete the bridging of a 10‐mm rat sciatic nerve defect in 21 days.

### 4.2 Intervention treatments of peripheral nerve repair by external stimulation

Intervention treatments by external stimulation can effectively promote nerve regeneration. For example, electrical and ultrasound stimulation are often applied during the rehabilitation of patients with PNI to promote the function recovery. Combining external stimulation with NGCs is an important development direction for nerve regeneration, which is of great importance for the treatment of thick nerve in large gap.

**FIGURE 6:**
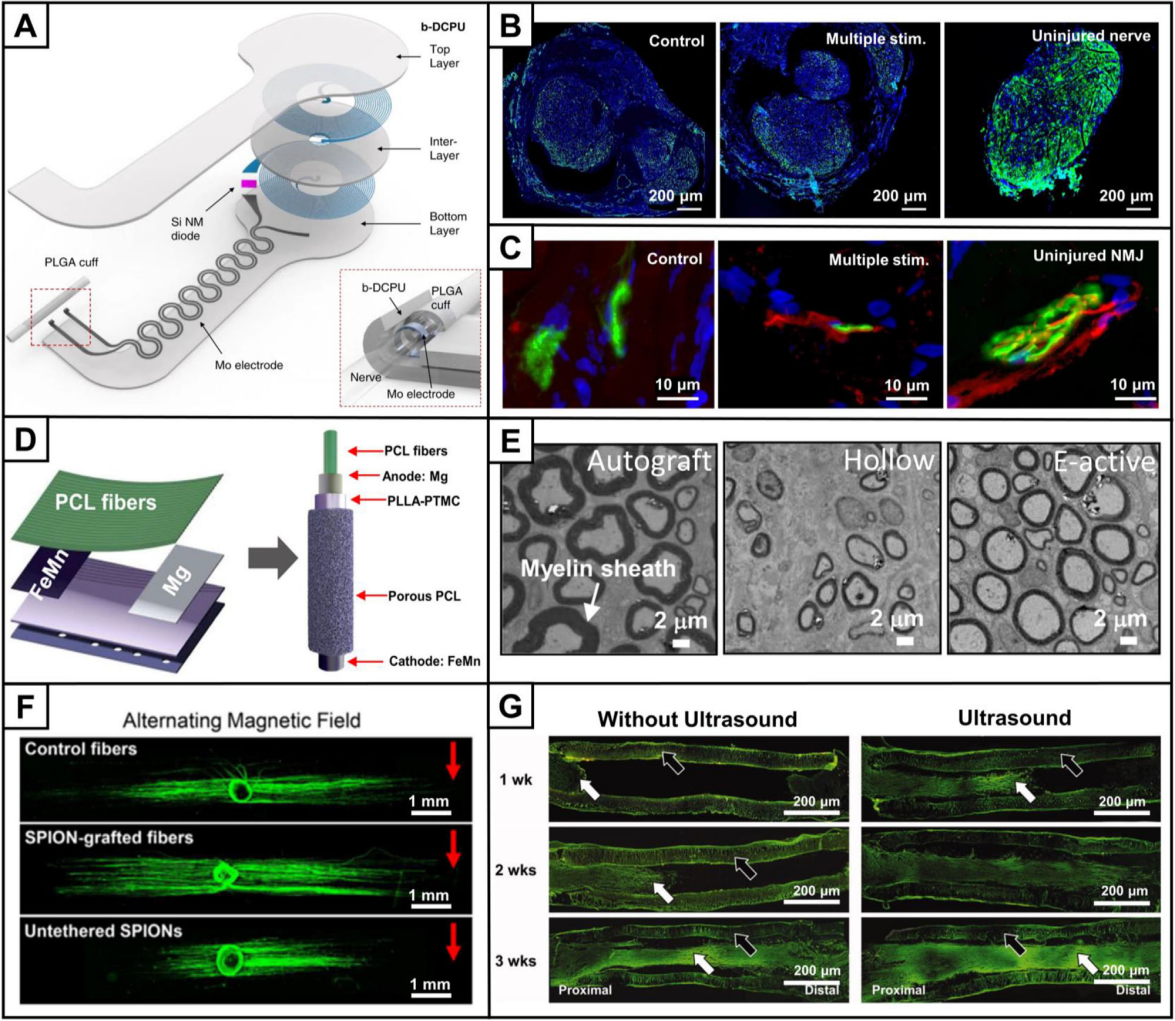
(A) Schematic illustration showing the design of an electrically active scaffold constructed from an inductive Mo coil and a radiofrequency diode (Si nanomaterial diode, Si NM diode), bioresorbable dynamic covalent polyurethane (b‐DCPU) layers, and a PLGA conduit. Fluorescence micrographs of the (B) regenerated nerves and (C) neuromuscular junction (NMJ) in the conduits without electrical stimulation (control group) and with multiple electrical stimulation (multiple stim. group) in a 10‐mm rat sciatic nerve injury model, respectively, as well as the uninjured nerve. The regenerated nerves in (B) were stained with Tuj1 (green), and the cell nuclei were stained with DAPI (blue). Double staining of the neuromuscular junction (NMJ) in (C) demonstrates significantly increased overlapping of pre‐ (neurofilament, red color) and postsynaptic (alpha‐bungarotoxin, green color) staining for the group with multiple episodes of distal nerve stimulation, indicating an enhanced number of NMJ and muscle reinnervation. Reproduced with permission.^[^ ^70^ ^]^ Copyright 2020, Springer Nature. (D) Schematic illustration of a biodegradable, self‐electrified, and miniaturized conduit. (E) TEM images of the middle sections of regenerated nerves when autograft, hollow conduit, and electroactive conduit group (E‐active) were implanted to repair a 10‐mm rat sciatic nerve injury, respectively. Reproduced with permission.^[^ ^71^ ^]^ Copyright 2020, The American Association for the Advancement of Science. (F) Fluorescence micrographs of DRG cultured on control fibers, superparamagnetic iron oxide nanoparticles (SPIONs)‐grafted fibers, and fibers incubated with untethered SPIONs in the alternating magnetic field condition. The neurites extending from DRG were stained with neurofilament (green). The red arrow indicates the side of the DRG closed to the alternating magnet. Reproduced with permission.^[^ ^76^ ^]^ Copyright 2021, Elsevier. (G) Longitudinal sections of the regenerated nerves (green) through NGC without and with ultrasound treatment in a 10‐mm rat sciatic nerve injury model, respectively. The white and black arrows indicate the regenerated nerve and the wall of the tube, respectively. Reproduced with permission.^[^ ^78^ ^]^ Copyright 2010, John Wiley & Sons

Since natural nerve tissue is electrically active, electrical signals can provide stimulation for nerve connectivity and cell growth to improve nerve regeneration. For example, by applying an external electrical stimulation to an electrically conductive scaffold, the axon extension was promoted.^[^ ^69^ ^]^ Electrical stimulation usually require a periodic treatment and a connection to an external voltage via a wire, which is inconvenient to use and carries the risk of infection. A bioabsorbable scaffold with wireless electrical stimulation was developed to improve the convenience and reduce the risk of infection, as shown in Figure 6A.^[^ ^70^ ^]^ The scaffold consisted of a radiofrequency power harvester constructed from an inductive Mo coil and a radiofrequency diode (Si nanomaterial diode), bioresorbable dynamic covalent polyurethane layers, and a PLGA conduit. Upon multiple electrical stimulation, both the electrophysiological activity and motor function were improved when repairing a 10‐mm rat sciatic nerve injury model. No significant difference was observed in the mature axon signals (green color) in the regenerated nerves with or without electrical stimulation (Figure 6B), while an increase in the functional innervation of the neuromuscular junctions was achieved upon electrical stimulation (Figure 6C). This scaffold was large and required an external equipment. Smaller, self‐powered implants are more attractive and convenient. A biodegradable conduit was embedded with a primary cell composed of Mg and Fe‐Mn alloy electrodes and showed electrically active property (Figure 6D).^[^ ^71^ ^]^ After implanting the conduit to repair a 10‐mm rat sciatic nerve injury, both the thickness of the regenerated myelin sheath (Figure 6E) and restoration of motor function were comparable to those in the autograft group. The detailed mechanism of electrical stimulation for promoting nerve regeneration is complex.^[^ ^72^, ^73^, ^74^ ^]^ Through electrical stimulation, the regeneration‐associated genes can be regulated, leading to the acceleration of axonal extension, remyelination of regenerating axons, and reinnervation of the muscle and target tissue.

Other external stimulations, such as magnetic field, ultrasound, and light, can also promote peripheral nerve regeneration through directly working with the nerve or serving as a trigger for on‐demand release of biological effectors from the NGCs. For example, magnetic particles were endocytosed by neuron cells and then transported to growth cones, generating a mechanical force under the action of a magnetic field to promote axonal extension of PC12 cells.^[^ ^75^ ^]^ In another study, DRG were cultured on control fibers, superparamagnetic iron oxide nanoparticles (SPIONs)‐grafted fibers, and fibers incubated with untethered SPIONs (Figure 6F).^[^ ^76^ ^]^ Under the stimulation of an alternating magnetic field, the DRG cultured on the SPIONs‐grafted fibers extended more and longer neurites on both sides, demonstrating that the magnetic field combined with the magnetic particle‐containing fibers could promote the nerve repair.

The mechanical stimulation and cavitation mechanism generated by ultrasound also play an important role in nerve regeneration.^[^ ^77^ ^]^ The mechanical strain generated by ultrasound can enhance enzyme activity and accelerate cell metabolism. As shown in Figure 6G, ultrasound intervention significantly accelerated the nerve regeneration in a 10‐mm rat sciatic nerve injury model.^[^ ^78^ ^]^ The intensity of the ultrasonic stimulation could affect the efficacy of nerve repair.^[^ ^79^ ^]^ The possible reason was that ultrasound stimulation at medium‐ and low‐intensity could improve cell membrane permeability, enhance substance transfer, and increase nutrient uptake, whereas a high‐intensity ultrasound stimulation may cause sound pressure and local high temperature, leading to the damage to cell membrane, cytoskeleton, and mitochondria.

Light stimulation has also shown its role in nerve regeneration by inhibiting the production of inflammatory factors, such as hypoxia inducible factor‐1α, tumor necrosis factor‐α, and interleukin‐1β, and increasing the expression of NGF and VEGF.^[80]^ As mentioned above, near‐infrared laser irradiation can also trigger the release of biological effectors from the conduit to promote nerve regeneration. The application of external stimulation to nerve regeneration is still in the basic research stage, so many challenges still need to be overcome before they can be applied in clinic. It is necessary to explore the mechanism of external stimulation on the nerve repair and to confirm the stage, frequency, duration, and intensity of the applied external stimulation. Improper application of the external stimulation may have unexpected effects on nerve repair and may even be counterproductive.

### 4.3 Multifunctional platform for the treatment, monitoring, and evaluation

Constructing NGCs with specific structures to provide physiochemical cues is important for modulating cell behavior and axon extension. The introduction of biological effectors and cellular components can endow bioactivity to the NGCs to further improve the deficiencies of the damaged microenvironment and promote nerve regeneration. In order to further promote nerve regeneration and advance clinical translation, the treatment efficacy of currently available tissue engineered NGCs still needs to further improve. The introduction of in situ imaging technique and external stimulation are attractive approaches to improve PNI repair. To maximize the therapeutic outcome, combining both of them with NGCs should be considered to enable a real‐time monitoring of the nerve regeneration process and thus guide the application of the different external stimulation at the right time and position, achieving the imaging guided treatment and regeneration. For example, a suitable amount of MRI contrast agents can be loaded in the NGCs to resolve the regeneration position of the nerve, guiding the application of external stimulation such as light for triggering the release of biological effectors to promote nerve repair. As another example, near‐infrared probes can be loaded in the NGCs for severing as not only imaging agents but also photothermal agents and drug carrier, enabling the guided, spatiotemporal controlled release of biological effectors for nerve repair. To achieve synergistic tissue regeneration, the appropriate conditions for the imaging process and the applied external stimulation, such as the amount and type of contrast agents in the NGCs, and the power density and duration of imaging and external stimulation, are important parameters need to be determined by in vitro and in vivo investigations. It will be promising for the prospect and development of clinical treatment of PNI by building an integrated, multifunctional platform by combination of NGCs, imaging technique, external stimulation, and other therapeutic methods for the treatment, monitoring, and evaluation.

## 5 CONCLUSIONS

The construction of tissue engineered NGCs by effectively integrating multiple guidance signals, such as topographical design, controlled delivery of biological effectors, and cellular component, is of great importance for peripheral nerve repair. The combination of imaging techniques with tissue engineered NGCs has achieved wide attention to allow for the monitoring of the regeneration progress. The introduction of therapeutic interventions upon external stimulation at specific stage of nerve repair under the guidance of imaging technique is also crucial for improving function recovery. In the future, the NGCs combined with multiple guidance signals can serve as an integrated platform for the treatment, monitoring, and evaluation of the nerve repair, enabling the repair of thick nerve in large gap and the translation from research to the clinic.

## CONFLICT OF INTEREST

The authors declare no competing financial interest.
